# Structure and photoluminescence of the TiO_2_ films grown by atomic layer deposition using tetrakis-dimethylamino titanium and ozone

**DOI:** 10.1186/s11671-015-0790-x

**Published:** 2015-02-28

**Authors:** Chunyan Jin, Ben Liu, Zhongxiang Lei, Jiaming Sun

**Affiliations:** Key Laboratory of Weak Light Nonlinear Photonics, Ministry of Education, School of Physics, Nankai University, Weijin Road 94, Tianjin, 300071 China; Air Force Aviation University, Nanhu Road No. 2222, Changchun, 130022 China

**Keywords:** Atomic layer deposition, Titanium dioxide, Photoluminescence, Crystal structure, Defects, XPS, 81.15.Gh, 72.20.Jv, 78.20.Ci

## Abstract

TiO_2_ films were grown on silicon substrates by atomic layer deposition (ALD) using tetrakis-dimethylamino titanium and ozone. Amorphous TiO_2_ film was deposited at a low substrate temperature of 165°C, and anatase TiO_2_ film was grown at 250°C. The amorphous TiO_2_ film crystallizes to anatase TiO_2_ phase with annealing temperature ranged from 300°C to 1,100°C in N_2_ atmosphere, while the anatase TiO_2_ film transforms into rutile phase at a temperature of 1,000°C. Photoluminescence from anatase TiO_2_ films contains a red band at 600 nm and a green band at around 515 nm. The red band exhibits a strong correlation with defects of the under-coordinated Ti^3+^ ions, and the green band shows a close relationship with the oxygen vacancies on (101) oriented anatase crystal surface. A blue shift of the photoluminescence spectra reveals that the defects of under-coordinated Ti^3+^ ions transform to surface oxygen vacancies in the anatase TiO_2_ film annealing at temperature from 800°C to 900°C in N_2_ atmosphere.

## Background

TiO_2_ has become a promising material in different applications for its large band gap [1], high refractive index [2,3], high dielectric constant [4,5], and highly active surface. In terms of photochemical properties, TiO_2_ is used in decomposition of water into hydrogen and oxygen [6] and served as a photocatalyst in solar cells [7]. Degradation of organic molecules is another active research topic, such as purification of waste water [8], disinfection in public [9], self-cleaning coating [10], corrosion-protection [11], and actively suppressed impact on tumor cells of rats illuminated by near-UV [12-14]. In addition, TiO_2_, as a semiconducting metal oxide, can be used as oxygen gas sensor to control the air/fuel mixture in car engines [15,16]. The high dielectric constant broadens the applications of TiO_2_ in electronics, such as capacitor and memory device. In our daily life, titanium dioxide pigment is almost used in every kind of paint because of its high refractive index. Moreover, pure TiO_2_ is non-toxic and easy-dispersive, and it can be used in food additive [17], in cosmetic products, as well as in pharmaceuticals [18].

Among extensive applications using physical and surface chemical properties of TiO_2_, the defects and the surface states of TiO_2_, which depend strongly on material preparation technologies, play an important role in its electrical, chemical, as well as optical properties. Therefore, selection of a well-controllable technology to engineer the defects in TiO_2_ will be crucial for specific application. Almost all viable physical and chemical deposition technologies have been adopted to prepare TiO_2_ thin films. Atomic layer deposition (ALD) has distinguished advantages over others for its precise thickness control, extremely conformal surface coating for nanostructures, large area uniformity, and low growth temperature [19-21]. Several precursors have been applied successfully for deposition of TiO_2_ by ALD processes. The common precursor, TiCl_4_, is a liquid with a moderate vapor pressure [22-27]. In the ALD process with H_2_O/H_2_O_2_ as oxidant, the corrosive by-products of HCl and residual TiCl_4_ are considered as a drawback. Same as Ti halide, TiI_4_ can also be served as another precursor [28-30] with relative less corrosive, compared to TiCl_4_. Recently, titanium alkoxides become promising precursors without corrosive halogen by-products, and research has been carried out on isopropoxide (Ti(O^i^Pr)_4_) and titanium ethoxide (Ti(OEt)_4_). Although high purity thin films can be grown at 300°C, the decomposition of precursor leads to an undistinguished ALD temperature window. In addition, titanium isopropoxide can be adopted as precursor in theory, but significant decomposition occurs at lower deposition temperature than that of the titanium ethoxide. Since the bond energy of metal-halide is much stronger than that of the metal-nitrogen bond, metal amide compounds are expected to have much higher reactivity with H_2_O, and therefore, tetrakis-(dimethylamino) titanium (TDMAT) and H_2_O have been used for ALD processes [31,32]. However, using H_2_O as oxidant has two main disadvantages: the water vapor exposure on TiO_2_ surface requires a very long purge time at the deposition temperature below 150°C [33], and the H_2_O-based ALD process brings impurities, such as hydroxyl groups (−OH) in the films [34,35]. The “dry” ALD process of TiO_2_ films using TDMAT and ozone (O_3_) may have more advantages, comparing to the TDMAT and H_2_O process. Only a few reports have been published concerning the TDMAT and O_3_ process [36,37], and the study on controlling the transformation of structure and defects has not yet been done in ALD TiO_2_ films. A comprehensive research on the thermal stability of the structure and defects in the ALD TiO_2_ film is crucial for controlling its electric and optical properties for different applications.

In this study, TiO_2_ films were deposited on silicon substrates by ALD technology using TDMAT and ozone process. The dependences of the growth rate, refractive index, and crystal structure and defects of the TiO_2_ films on the growth temperatures are investigated in details by optical ellipsometry, X-ray diffraction (XRD), photoluminescence (PL), and X-ray photoelectron spectroscopy (XPS). Annealing processes were performed comparably on two as-grown TiO_2_ films with amorphous and anatase phase structures, respectively. Thermal stability of the structure and defects in the as-grown TiO_2_ films and those annealed at different temperatures were studied by PL spectroscopy in conjunction with XRD and XPS analysis. Amorphous, anatase, and rutile TiO_2_ films were prepared at different ALD growth temperatures or by annealing at different temperatures. The PL spectra show a red band at 600 nm and a green band at around 515 nm from the defects in anatase TiO_2_ films. It was shown that the red band has a strong correlation with the defects associated with under-coordinated Ti^3+^ ions and the green band is related to the oxygen vacancies on (101) surface of anatase TiO_2_ films. The blue shift of the PL spectra indicates that the defects in anatase TiO_2_ film undergo a transformation from under-coordinated Ti atoms to surface oxygen vacancies with increasing the annealing temperature from 800°C to 900°C in N_2_ atmosphere.

## Methods

TiO_2_ films were deposited on 4-in. (100) oriented n-type silicon wafers by a small chamber ALD system (Cambridge NanoTech Savannah 100, Cambridge NanoTech Inc., Cambridge, MA, USA) using TDMAT and O_3_. The evaporation temperature of the TDMAT source was kept at 60°C, and the precursor delivery lines were heated at 150°C. O_3_ was generated from high purity O_2_ (99.999%) through an ozone generator with an O_2_/O_3_ flow of 500 sccm and O_3_ concentration of 36 mg/L. High purity nitrogen gas (99.999%) was used as a carrying and purging gas with a flow rate of 20 sccm. Before the film deposition, the Si wafer was cleaned through the standard process of Radio Corporation of America (RCA), followed by a final cleaning in diluted HF solution. TiO_2_ samples with 1,000 ALD cycles were deposited at different substrate temperatures varying from 75°C to 400°C. One TiO_2_ deposition cycle consists of 0.5 s TDMAT pulse time, 5 s N_2_ purge, 1.8 s O_2_/O_3_ pulse time, and 9 s N_2_ purge, respectively. The thermal stability of the structures of ALD TiO_2_ films was studied by annealing two as-grown ALD samples with different initial structures; one is an amorphous TiO_2_ film grown at low substrate temperature of 165°C, and the other is an anatase TiO_2_ film grown at 250°C. The annealing treatment was taken at different temperatures from 250°C to 1,150°C in a flowing N_2_ atmosphere for 1 h.

The crystallinity of the TiO_2_ films was characterized by XRD with Cu K_α_ radiation. The thickness and refractive index of the TiO_2_ films on Si substrates were measured by an ellipsometer with a 632.8-nm He-Ne laser beam at an incident angle of 69.8°. The film growth per cycle was calculated by dividing the film thickness with the total number of ALD cycles. PL spectra of the TiO_2_ films were measured at room temperature under the excitation of the 266-nm line of a pulsed diode pumped Q-switch solid state laser (CryLas DX-Q, CryLaS GmbH, Berlin, Germany). The PL signal was collected by a 1/2 meter monochromator and detected by a photomultiplier (model H7732-10, Hamamatsu Corporation, Shimokanzo, Iwata, Japan) connected to a computer-controlled Keithley 2010 multimeter (Keithley Instruments Inc., Cleveland, OH, USA). XPS measurement was performed in a Kratos Axis Ultra DLD spectrometer (Kratos Analytical Ltd, Britain). Monochromatized Al-Kα X-ray source (*h*_*γ*_ = 1,486.6 eV) was utilized to excite TiO_2_ thin films. X-ray photoelectron spectra were measured from the surface of the TiO_2_ samples annealed at 350°C, 600°C, 800°C, 850°C, and 1,000°C. For comparison, XPS was measured from the sample annealed at 1,000°C after removing 3 nm of the surface layer by Ar^+^ ion sputtering. The Ar^+^ ion sputtering was performed over an area of 2 × 2 mm^2^, using an ion current of about 100 mA. The binding energy of each spectrum was calibrated by using the standard energy of carbon C1s peak at 284.6 eV.

## Results and discussion

**Figure 1:**
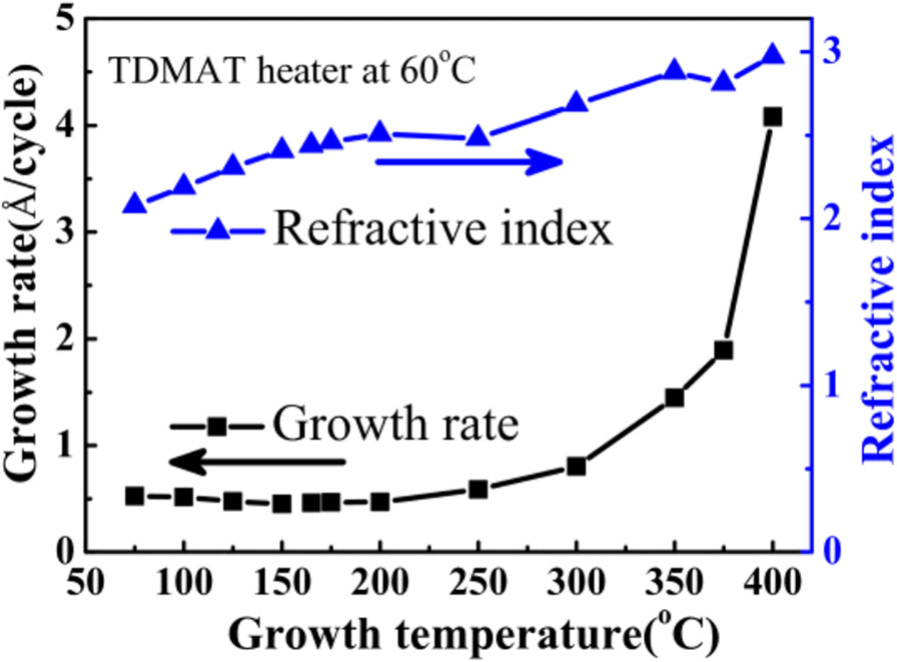
**Dependences of the growth per cycle and refractive index on the growth temperature.** The TiO_2_ films were deposited on Si (100) substrates with 1,000 ALD cycles.

Figure 1 shows the dependences of the growth per cycle and the refractive index of the TiO_2_ films on the growth temperature. Initially, the growth rate of the TiO_2_ films decreases from 0.52 to 0.45 Å/cycle with increasing temperature from 75°C to 100°C, then a saturated growth window appears at the growth temperature from 100°C to 250°C, with a stable self-limiting growth rate of 0.46 Å/cycle. Further increasing the growth temperature above 300°C, the growth rate strongly increases. The growth rate in Figure 1 is consistent with the results in ref. [37] in the same temperature range, which showed an ALD temperature window of 150°C to 225°C and a deposition rate of 0.44 ± 0.15 Å/cycle, respectively.

The dependence of the growth per cycle on the growth temperature in this O_3_-based process is different from that of the TDMA and H_2_O process reported in ref. [31,32,38]. In the H_2_O-based process, where -OH groups are the reactive sites, the deposition rate decreases with increasing the growth temperature from 80°C to 350°C. The growth rate of TiO_2_ at growth temperature below 150°C could be strongly influenced by the purging time of H_2_O vapor. The reported results on the growth rate per cycle are controversial using TDMAT and H_2_O process, depending on the ALD systems used by different groups. Lim and Kim observed a narrow ALD window between 120°C and 150°C in the TDMAT and H_2_O process [31], whereas other reports showed a decrease of the growth rate with increasing temperature from 150°C up to 350°C, without distinguished saturated growth temperature window [32,39]. This is probably due to the insufficient evacuation of the residual H_2_O vapor in the growth chamber [40]. The decrease of the growth rate in the H_2_O-based process with increasing the growth temperature was probably caused by the strong thermal desorption of the intermediate product mediated by -OH group adsorption on the surface, as proposed in ref. [32]. The possible chemical reaction of the surface species with -OH groups is:

$$ {\mathrm{TiO}}_2\hbox{-} \mathrm{O}\hbox{-} \mathrm{T}\mathrm{i}{\left[\mathrm{N}{\left({\mathrm{CH}}_3\right)}_2\right]}_3*+{\mathrm{TiO}}_2\hbox{-} \mathrm{O}\mathrm{H}*\to {\mathrm{TiO}}_2\hbox{-} \mathrm{O}*\hbox{-} {\mathrm{TiO}}_2+\mathrm{HOTi}{\left[\mathrm{N}{\left({\mathrm{CH}}_3\right)}_2\right]}_3\uparrow . $$

On the contrary, in the O_3_-based process, the desorption of intermediate products is suppressed without the surface adsorption of the -OH groups from H_2_O vapor; therefore, a wider ALD saturated growth temperature window from 100°C to 250°C was observed, and the growth rate shows a strong increase of from 0.58 to 4.08 Å/cycle with increasing the growth temperature from 250°C to 400°C. The strong increase of the growth rate is due to the chemical vapor deposition (CVD) process which is related to the strong thermal decomposition of the TDMAT precursor [41,42] at temperature above 250°C.

**Figure 2:**
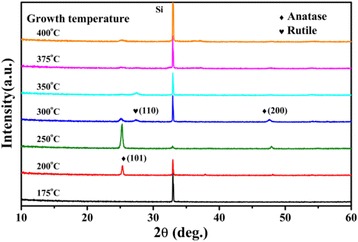
**XRD patterns of the TiO**_**2**_ **films deposited at different temperatures.**

Figure 2 shows the XRD patterns of the TiO_2_ films deposited at different growth temperatures from 175°C to 400°C. Initially, the films deposited at temperatures below 175°C are amorphous. With increasing growth temperature from 200°C to 250°C, the films show anatase crystal phase, with the (101) and (200) peaks in the diffraction patterns. The intensity of the anatase (101) peak reaches a maximum at the growth temperature of 250°C and then decreases dramatically to 300°C, with an emergence of a weak (110) peak from rutile TiO_2_. At growth temperature above 250°C, the growth mode of the films changes to fast CVD mode, the fast deposition rate causes a strong degradation of the crystallinity of the TiO_2_ film, as shown by the decrease of the diffraction peaks in the XRD patterns at 300°C to 400°C. Despite of this, very weak (101) peak from anatase TiO_2_ and (110) peak from rutile TiO_2_ are observed in the XRD patterns, indicating the formation of a small among of rutile TiO_2_ in the films. As it was reported that rutile TiO_2_ is the stablest and densest structure of TiO_2_ with a mass density of 4.25 g/cm^3^, while the anatase TiO_2_ is a metastable and less dense structure, with a smaller density of 3.894 g/cm^3^ [43]. The increasing tendency of the refractive index from 2.07 to 2.97 in Figure 1, which can be interpreted by the structure change in the films with increasing the growth temperature, is probably due to the film densification with the change from amorphous to anatase as well as the formation of rutile phase [44].

**Figure 3:**
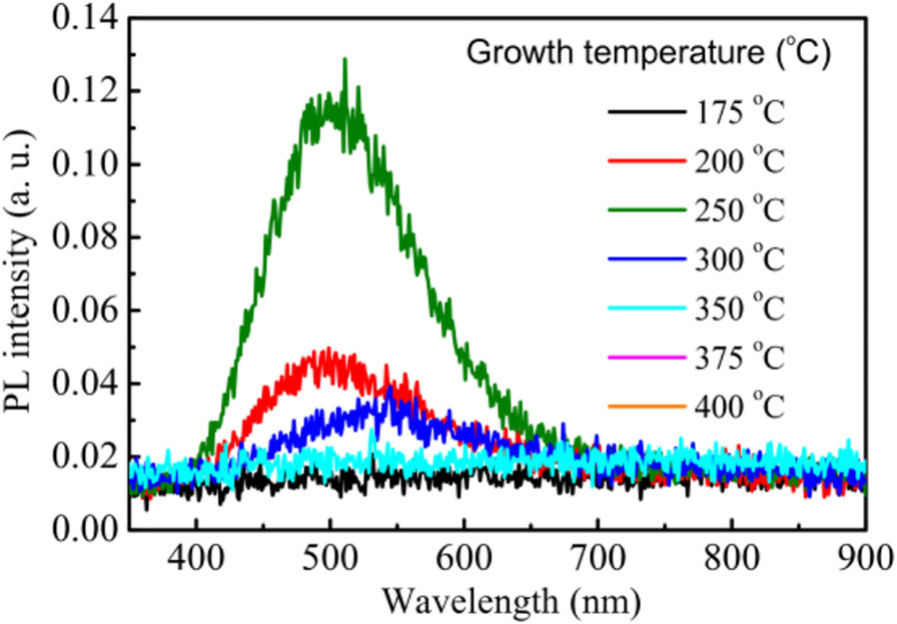
**Room-temperature PL spectra from TiO**_**2**_ **films grown at different temperatures.** The spectra were taken under excitation of a 266 nm laser.

**Figure 4:**
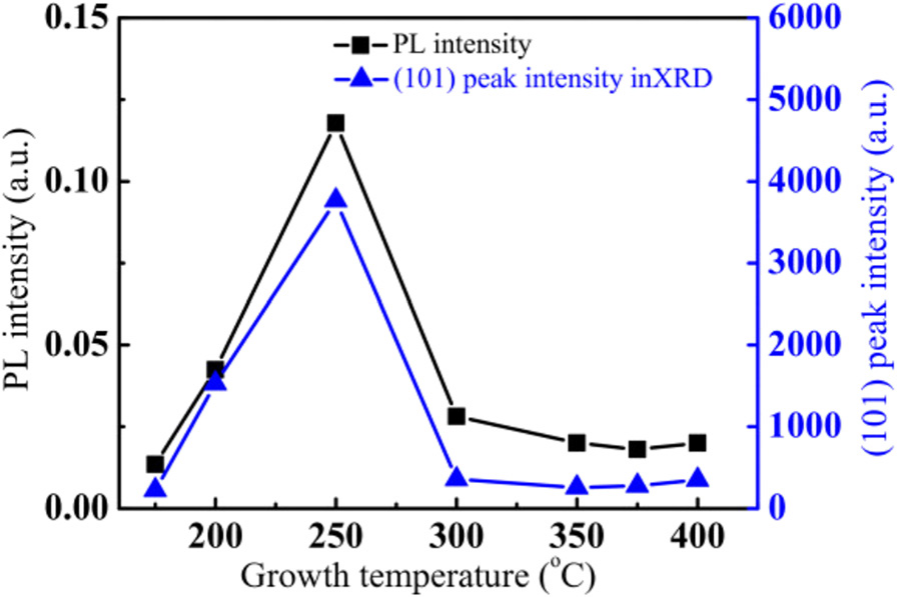
**Dependences of the PL intensity and the anatase (101) peak intensity in XRD patterns on the growth temperature.**

The change of defects in the TiO_2_ films was characterized by measuring the room-temperature PL spectra at different growth temperatures. As it was shown in Figure 3, no PL emission was detected from the amorphous TiO_2_ films grown below 175°C. A green PL band at around 500 nm was observed from the TiO_2_ films with anatase phase grown at temperatures from 200°C to 300°C. In order to study the correlation between the PL and the structure change in the films, the dependences of PL peak intensity and the intensity of (101) anatase peak from the XRD patterns on the growth temperature are plotted together in Figure 4. The PL intensity increases with increasing substrate temperature, reaches a maximum at 250°C, and then decreases strongly at higher growth temperature, which is similar to the growth temperature dependence of the (101) anatase peak intensity in the XRD patterns. This similarity indicates that the defects related to the green PL band are probably located on the (101) oriented surface of the anatase TiO_2_ crystals. Finally, the strong quenching of the PL intensity at growth temperature over 250°C is probably due to the degradation of the anatase crystallinity by CVD, which causes an increase of the non-radiative recombination centers in the films.

**Figure 5:**
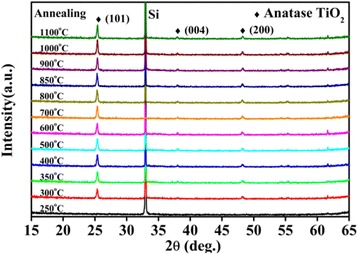
**XRD patterns of the TiO**_**2**_ **films annealed at different temperatures in N**_**2**_ **atmosphere.** The as-grown TiO_2_ film is amorphous deposited at a substrate temperature of 165°C.

The thermal stability of the structures in ALD TiO_2_ films was studied by annealing two as-grown samples with different initial structures in N_2_ atmosphere; one is an amorphous TiO_2_ film grown at low substrate temperature of 165°C, and the other is an anatase TiO_2_ film grown at 250°C. Figure 5 shows the XRD patterns from the TiO_2_ films after annealing the as-grown amorphous sample at different temperatures in N_2_ atmosphere for 1 h. Initially, the films are still amorphous at lower annealing temperature below 250°C, then they crystallize to anatase TiO_2_ in a very wide annealing temperature range from 300°C to 1,100°C, as shown in the emergence of the (101), (004), and (200) peaks of anatase TiO_2_ in the XRD patterns in Figure 5.

**Figure 6:**
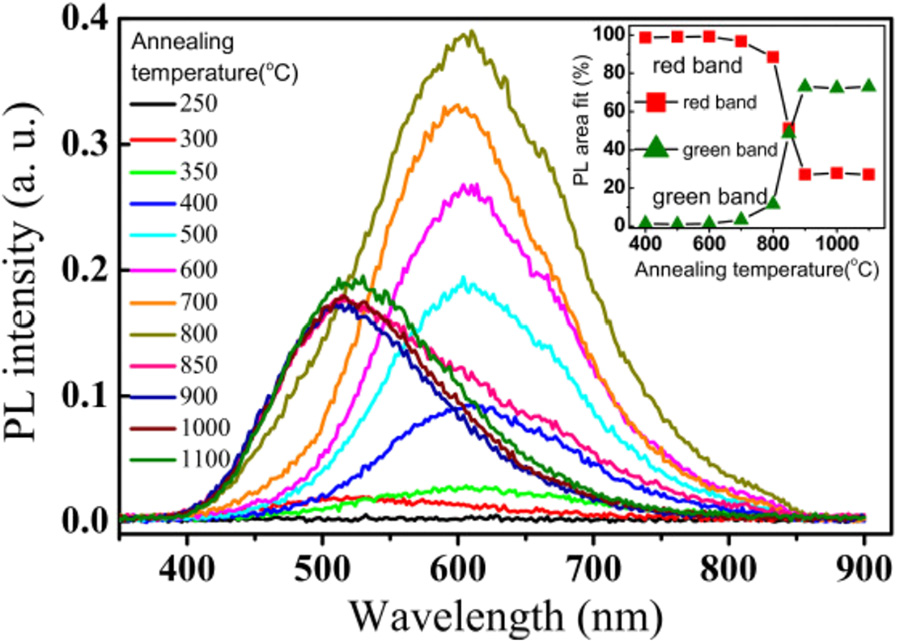
**Room-temperature PL spectra of the TiO**_**2**_ **films annealed at different temperatures in N**_**2**_ **atmosphere.** The as-grown TiO_2_ film is amorphous deposited at a substrate temperature of 165°C. The insert is the areal percentages of the red emission band at 600 nm and the green emission band at 515 nm, which are derived from multiple-peak Gaussian fitting of the PL spectra, respectively.

Thermal stability of the defects in the annealed TiO_2_ films in Figure 5 was characterized by the PL spectra in Figure 6. The PL spectra can be decomposed into a red band at 600 nm and a green band at 515 nm. The insert shows the areal percentages of the red emission and the green band, which are derived from multiple-peak Gaussian fitting of the PL spectra, respectively. In conjunction with the XRD patterns in Figure 5, no PL emission is observed from the amorphous TiO_2_ films at annealing temperature lower than 250°C. The red PL band was observed from the defects in the anatase TiO_2_ films, which were obtained by annealing the as-grown amorphous films at annealing temperatures from 300°C to 800°C. The intensity of the red band increases to a maximum for annealing temperature from 300 to 800°C, and then, it decreases strongly with increasing temperature. For annealing temperature from 800°C to 900°C, the green band appears with the decrease of the red band and the PL spectra undergo a crossover from the red band-dominated emission to the green band-dominated emission as shown in the insert. At even higher annealing temperatures from 900°C to 1,100°C, the PL spectra are dominated by the green band with a saturated intensity.

**Figure 7:**
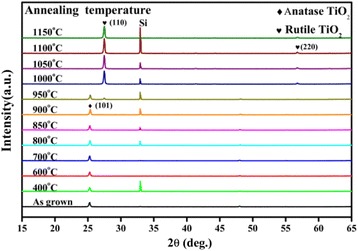
**XRD patterns of TiO**_**2**_ **films after annealing at different temperatures in N**_**2**_ **atmosphere.** The as-grown TiO_2_ film is anatase deposited at a substrate temperature of 250°C.

Figure 7 is the XRD patterns from the TiO_2_ films after annealing at different temperatures in N_2_ atmosphere for 1 h, in which the as-grown TiO_2_ film was initially in anatase phase deposited at a substrate temperature of 250°C. Initially, the annealed TiO_2_ films still keep anatase phase in a wide annealing temperature range from 400°C to 900°C, and then, a clear transition from anatase to rutile phase was observed in the annealing temperature range from 950°C to 1,000°C. Finally, the anatase films change to rutile TiO_2_ phase at elevated annealing temperatures above 1,000°C.

**Figure 8:**
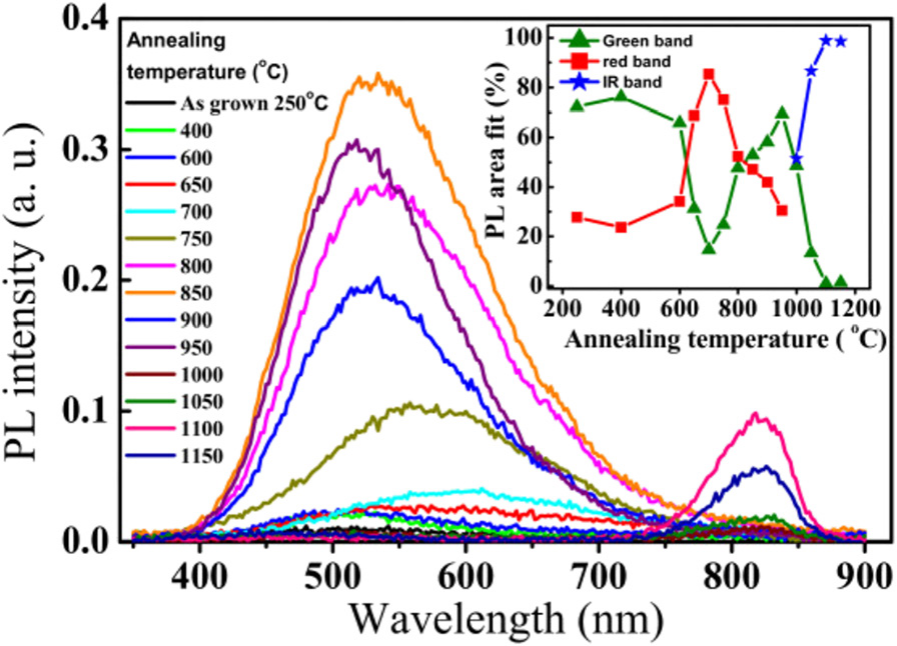
**Room-temperature PL spectra of the TiO**_**2**_ **films annealed at different temperatures in N**_**2**_ **atmosphere.** The as-grown TiO_2_ film is anatase deposited at a substrate temperature of 250°C. The insert is the areal percentages of the red emission band at 615 nm, the green emission band at 510 nm, and the near-infrared peak at 820 nm, which are derived from multiple-peak Gaussian fitting of the PL spectra, respectively.

Figure 8 shows the room-temperature PL spectra for the TiO_2_ samples in Figure 7 annealed at different temperatures, in which the as-grown film is in anatase TiO_2_ phase. The broadband PL emission from the anatase TiO_2_ films can be divided into two components with the red peak centered at 600 nm and the green peak centered at 515 nm. Gaussian fitting of the PL spectra was performed using the parameters of the red and green peaks derived from Figure 6. The insert shows the areal percentage of the red and the green components, respectively. The PL spectrum from the as-grown anatase TiO_2_ film contains 24% of the red component and 76% of the green component. With increasing annealing temperature from 400°C to 700°C, the PL intensity of the sample increases slightly, the red component of the spectra increases from 24% to 85%, while the green component reduces from 76% to 15%; therefore, the PL spectra exhibit a red shift. For increasing the annealing temperature from 700°C to 850°C, a strong increase of the PL intensity was observed, the red component decreases from 85% to 47%, while the green component rises from 15% to 53% in the spectra, the relative increase of the green component causes a blue shift of the PL peak. Further increasing the annealing temperature from 850°C to 1,000°C, the PL peaks in the visible spectral range decrease dramatically due to the transition from the anatase to rutile TiO_2_ phase. Finally, the PL spectra show a near-infrared peak at 820 nm from the defects in rutile TiO_2_ formed at elevated annealing temperatures above 1,000°C.

**Figure 9:**
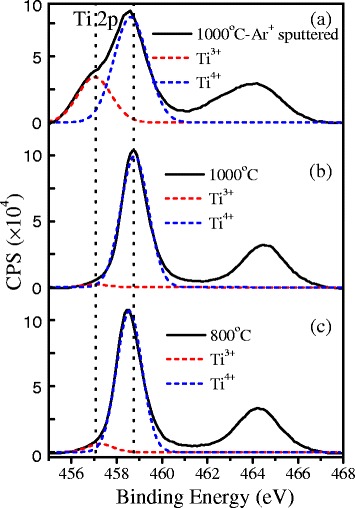
**XPS spectra of Ti 2p states from the TiO**_**2**_ **films.** Samples were annealed at 1,000°C after removing 3-nm surface layer by Ar^+^ ion sputtering **(a)**; annealed at 1,000°C without Ar^+^ ion sputtering **(b)**; and annealed at 800°C without Ar^+^ ion sputtering **(c)**. The dashed curves are multiple-peak Gaussian fitting of the Ti 2p_3/2_ peak with two components from the valence states of Ti^4+^ (blue) and Ti^3+^ (red).

XPS spectroscopy was studied after annealing the as-grown amorphous TiO_2_ films at different temperatures. Figure 9 shows the XPS peaks of Ti 2p_3/2_ and Ti 2p_1/2_ from the TiO_2_ film annealed at 1,000°C after removing 3-nm surface layer by Ar^+^ ion sputtering (a), the film annealed at 1,000°C without Ar^+^ ion sputtering (b), and the film annealed at 800°C without Ar^+^ ion sputtering (c). The binding energies of Ti 2p_3/2_ and Ti 2p_1/2_ peaks of Ti^4+^ ions in the sample annealed at 1,000°C are located at about 458.75 and 464.48 eV, respectively. After the removal of 3-nm surface layer by Ar^+^ ion sputtering, the Ti 2p_3/2_ peak shifts to lower energy at 458.54 eV and a shoulder peak at a lower energy of 457.0 eV appears. Multiple-peak Gaussian fitting of the spectrum indicates that the peak at lower energy belongs to the valence state of Ti^3+^ ions in the TiO_2_ film, which are formed by Ar^+^ ion sputtering, as reported in ref. [32,45,46]. The presence of the Ti^3+^ states in the films causes a small shift of the 2p_3/2_ peak of Ti^4+^ ions to lower energy compared to the un-sputtered one in Figure 9b. As a consequence, comparing the 2p_3/2_ peak of Ti^4+^ in the sample annealed at 800°C (c) with the one annealed at 1,000°C (b), a slight shift of Ti 2p_3/2_ peak from 458.75 to lower energy of 458.46 eV was also observed for the sample annealed at 800°C (c). This suggests that a small amount of trivalent Ti^3+^ ions exist in the sample annealed at 800°C. The relative concentration of the Ti^3+^ ions with respect to the total Ti atoms in the annealed anatase TiO_2_ films can be calculated from the integrated intensity of the 2p_3/2_ peak of Ti^3+^ ions (red dashed peak), which was derived by multiple-peak Gaussian fitting of the Ti 2p_3/2_ peak of the XPS, as shown by the dashed curves in Figure 9.

**Figure 10:**
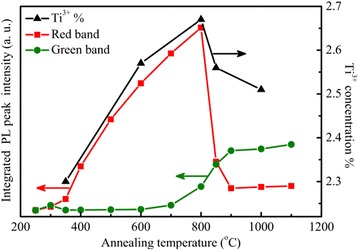
**Calculated concentration of Ti** ^**3+**^ **and the integrated PL intensity.** The calculated concentration of Ti^3+^ from XPS analysis (black triangles) and the integrated PL intensity from the red (red squares) and green (green dots) bands is derived from the Gaussian fitting of the XPS and PL spectra of the TiO_2_ films after annealing at different temperatures. The solid lines are for a guide of eyes.

In order to clarify the correlation between the photoluminescence and the defects related to the under-coordinated Ti^3+^ ions in the annealed TiO_2_ films, the integrated PL intensity of the red and green peaks as well as the percentage of Ti^3+^ in the films are plotted together as functions of the annealing temperature in Figure 10, in which the integrated PL intensity of the red and green peaks is derived by multiple-peak Gaussian fitting of the PL spectra in Figure 6. The integrated intensity of the green band was low at annealing temperature below 700°C, it increases from 700°C to 900°C, and then saturated at annealing temperature above 900°C. No obvious correlation was observed between the PL intensity of the green band and the Ti^3+^ ion concentration. The dependence of the integrated PL intensity of the red band on the annealing temperature shows a thermal behavior quite similar to the change of the Ti^3+^ ion concentration. Both of them increased with increasing annealing temperature from 300°C to 800°C, after reaches a maximum at 800°C and then decreases dramatically at annealing temperature varied from 800°C to 900°C. This similarity suggests that the red band may have a strong correlation with the defects associated with the under-coordinated Ti^3+^ ions in anatase TiO_2_.

Olson et al. [47] calculated the energies for creation of various defects, such as oxygen vacancy and Ti^4+^ and Ti^3+^ interstitials in TiO_2_. The formation energy is 24.10 eV (*E*_1_) for oxygen vacancy, which is higher than that for Ti^3+^ interstitial (*E*_2_ = −40.5 eV) and Ti^4+^ interstitial (*E*_3_ = −77.23 eV). The negative values of *E*_2_ and *E*_3_ indicate that the formation of the defects of under-coordinated Ti^3+^ is energetically favorable. Therefore, defects of Ti^4+^ interstitials and under-coordinated Ti^3+^ may form in higher priority at low annealing temperature during the crystallization of anatase crystal TiO_2_ from the as-grown amorphous film. Thus, the red band associated with electron traps of under-coordinated Ti^3+^ dominates the PL spectra at low annealing temperature in Figure 6. The defects of under-coordinated Ti^3+^ can be created with the removal of oxygen atoms by annealing in an inert N_2_ atmosphere or by Ar^+^ ion sputtering. The removal of oxygen atom can create lone pair electrons to two neighboring Ti^4+^, and then, electrons will reduce Ti^4+^ to Ti^3+^. This is confirmed by our XPS study in Figure 9.

Concerning the green PL band from the anatase TiO_2_ films, it dominates the PL spectra from the anatase TiO_2_ films grown at 250°C or the anatase TiO_2_ films which have undergone an annealing process at a high temperature above 850°C. This suggests that origin of the green band is probably from the relative stable surface oxygen vacancies on anatase TiO_2_ films. The strong correlation of the green band PL intensity with the intensity of (101) peak in XRD patterns of ALD grown TiO_2_ films in Figure 4 reveals that the green PL peak is related to the defects located on (101) surface in the anatase phase. Shi et al. and Mercado et al. [48,49] studied the PL emission from TiO_2_ nanocrystals, and they also draw the same conclusion that the green band emission is related to oxygen vacancies on exposed (101) surfaces of anatase TiO_2_ nanocrystals. Since the Ti^3+^ ions are unstable, as shown by the dependence of the Ti^3+^ ion density on the annealing temperature in Figure 10, the defects of under-coordinated Ti^3+^ ions can be annealed out, immigrate, and transform into stable surface oxygen vacancies on the anatase TiO_2_ films at high annealing temperature from 800°C to 900°C. Figure 6 shows a transition from the red band-dominated PL to the green band-dominated emission with increasing the annealing temperature from 800°C to 900°C. This reveals that some of the under-coordinated Ti^3+^ ions can transform into stable surface oxygen vacancies at high annealing temperature. This causes an increase of the intensity of the green PL band at annealing temperature from 800°C to 900°C. Since only the stable surface oxygen vacancies are preserved at elevated temperature [50]. Finally, the PL spectra are dominated by the green band with a saturation intensity at annealing temperatures above 900°C, as it is shown in Figures 6 and 10. This is also in accordance with the conclusion in ref. [51] that the green-emitting defects are oxygen vacancies located on the surface of anatase TiO_2_ films.

**Figure 11:**
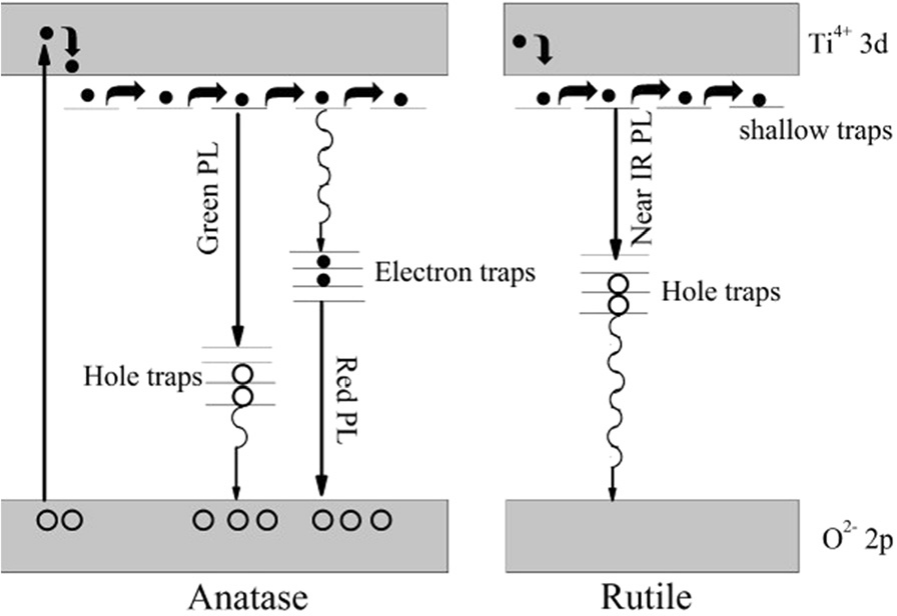
**Models for photoluminescence from the electronic transitions of trap states in anatase and rutile TiO**_**2**_ **.**

From the results of this study and the comprehensive study of the luminescent defects in TiO_2_ nanocrystals in ref. [48-51], the proposed model for PL in the ALD TiO_2_ films is illustrated in Figure 11. After electrons are excited from the valence band to the conduction band of TiO_2_, some electrons are captured by the electron traps associated with under-coordinated Ti atoms, which located at 0.7 to 1.6 eV below the conduction band edge. Radiative recombination of the electrons trapped around under-coordinated Ti atoms with the holes in the valence band contributes to the red band at around 600 to 620 nm. In addition, the green band at around 500 to 520 nm may be from the radiative recombination of free electrons with holes trapped around surface oxygen vacancies, which were located at 0.7 to 1.4 eV above the valence band edge. In addition, the near-infrared emission band at around 820 nm is from the defects in rutile TiO_2_, which are related to the radiative recombination of electrons in conduction band with hole traps on the (110) and (110) facets of oxygen vacancies [49].

## Conclusions

TiO_2_ films were grown on silicon substrates by ALD using TDMAT precursor and ozone. A wide ALD growth window was observed between 100°C and 250°C with a self-limiting saturated growth rate of 0.46 Å/cycle. The film is amorphous at the growth temperatures of 165°C and then exhibits anatase crystal phase at the growth temperatures of 250°C. The initial amorphous TiO_2_ sample crystallizes to anatase phase with annealing temperature from 300°C up to 1,100°C, while the initial anatase TiO_2_ film transfers to rutile phase at elevated annealing temperature above 950°C. Photoluminescence spectra from the defects in the anatase TiO_2_ films contain a red band at 600 nm and a green band at 515 nm. XPS and XRD studies indicate that the red band has a strong correlation with the defects of under-coordinated Ti^3+^ ions and the green band is related to the oxygen vacancies located on the (101) surface of the anatase TiO_2_ films. The blue shift of the photoluminescence reveals that the defects in anatase TiO_2_ film undergo a transition from under-coordinated Ti atoms to surface oxygen vacancies with increasing annealing temperature from 800°C to 900°C in N_2_ atmosphere.

## Abbreviations

ALD: atomic layer deposition
PL: photoluminescence
TDMAT: tetrakis-(dimethylamino) titanium
XRD: X-ray diffraction
XPS: X-ray photoelectron spectroscopy
RCA: Radio Corporation of America
CVD: chemical vapor deposition
